# Diazepam modulates anterior cingulate glutamate levels in people at clinical high-risk for psychosis

**DOI:** 10.1093/ijnp/pyaf078

**Published:** 2025-12-12

**Authors:** Amanda Kiemes, Nicholas R Livingston, Paulina B Lukow, Samuel Knight, Luke Jelen, Thomas Reilly, Aikaterini Dima, Maria A Nettis, David J Lythgoe, Cecilia Casetta, Alice Egerton, Thomas Spencer, Andrea De Micheli, Paolo Fusar-Poli, Anthony A Grace, Steven C R Williams, Philip McGuire, Cathy Davies, James M Stone, Gemma Modinos

**Affiliations:** Department of Psychological Medicine, Institute of Psychiatry, Psychology and Neuroscience, King’s College London, London, United Kingdom; Department of Psychological Medicine, Institute of Psychiatry, Psychology and Neuroscience, King’s College London, London, United Kingdom; Department of Psychological Medicine, Institute of Psychiatry, Psychology and Neuroscience, King’s College London, London, United Kingdom; Institute of Cognitive Neuroscience, University College London, London, United Kingdom; Department of Psychological Medicine, Institute of Psychiatry, Psychology and Neuroscience, King’s College London, London, United Kingdom; Department of Psychological Medicine, Institute of Psychiatry, Psychology and Neuroscience, King’s College London, London, United Kingdom; Department of Psychiatry, University of Oxford, Oxford, United Kingdom; South London and Maudsley NHS Foundation Trust, London, United Kingdom; Department of Psychosis Studies, Institute of Psychiatry, Psychology and Neuroscience, King’s College London, London, United Kingdom; South London and Maudsley NHS Foundation Trust, London, United Kingdom; Department of Psychological Medicine, Institute of Psychiatry, Psychology and Neuroscience, King’s College London, London, United Kingdom; Department of Neuroimaging, Institute of Psychiatry, Psychology and Neuroscience, King’s College London, London, United Kingdom; South London and Maudsley NHS Foundation Trust, London, United Kingdom; Department of Psychosis Studies, Institute of Psychiatry, Psychology and Neuroscience, King’s College London, London, United Kingdom; Department of Psychosis Studies, Institute of Psychiatry, Psychology and Neuroscience, King’s College London, London, United Kingdom; Department of Psychosis Studies, Institute of Psychiatry, Psychology and Neuroscience, King’s College London, London, United Kingdom; Department of Psychosis Studies, Institute of Psychiatry, Psychology and Neuroscience, King’s College London, London, United Kingdom; Department of Psychosis Studies, Institute of Psychiatry, Psychology and Neuroscience, King’s College London, London, United Kingdom; OASIS Service, South London and the Maudsley NHS Foundation Trust, London, United Kingdom; Department of Brain and Behavioural Sciences, University of Pavia, Pavia, Italy; Department of Psychiatry and Psychotherapy, Ludwig-Maximilian-University Munich, Munich, Germany; Departments of Neuroscience, Psychiatry and Psychology, University of Pittsburgh, Pittsburgh, PA, United States; Department of Neuroimaging, Institute of Psychiatry, Psychology and Neuroscience, King’s College London, London, United Kingdom; Department of Psychiatry, University of Oxford, Oxford, United Kingdom; Department of Psychosis Studies, Institute of Psychiatry, Psychology and Neuroscience, King’s College London, London, United Kingdom; Brighton and Sussex Medical School, University of Sussex, Brighton, United Kingdom; Department of Psychological Medicine, Institute of Psychiatry, Psychology and Neuroscience, King’s College London, London, United Kingdom; MRC Centre for Neurodevelopmental Disorders, King's College London, London, United Kingdom

**Keywords:** schizophrenia, benzodiazepines, clinical high risk for psychosis, Glx, magnetic resonance spectroscopy

## Abstract

**Objective:**

Preclinical evidence suggests that modulating neural excitation through administration of diazepam, a positive allosteric modulator of GABA_A_ receptors, can prevent the emergence of behavioral and neurobiological alterations relevant to psychosis in adulthood.

**Design and Participants:**

Here, we examine this neurochemical mechanism in individuals at clinical high risk for psychosis in a randomized, double-blind, placebo-controlled crossover study. Twenty-four individuals (15 female and 9 male) aged 18-35 were scanned twice using proton magnetic resonance spectroscopy to measure anterior cingulate cortex Glx (glutamate and glutamine) levels, once after a single dose of diazepam (5 mg) and once after placebo.

**Results:**

Mixed-effects model analyses revealed that diazepam reduced anterior cingulate cortex Glx levels compared to placebo (*t*(20.8) = −2.14, *P* = .04). The effect of diazepam on Glx levels was greater in older individuals at clinical high risk for psychosis (*t*(12) = −4.36, *P* = .001).

**Conclusion:**

These findings suggest that pharmacological modulation of GABA_A_ receptors can alter Glx changes in and support a novel therapeutic mechanism of benefit for individuals at clinical high risk of psychosis.

## Graphical Abstract

**Graphical Abstract:**
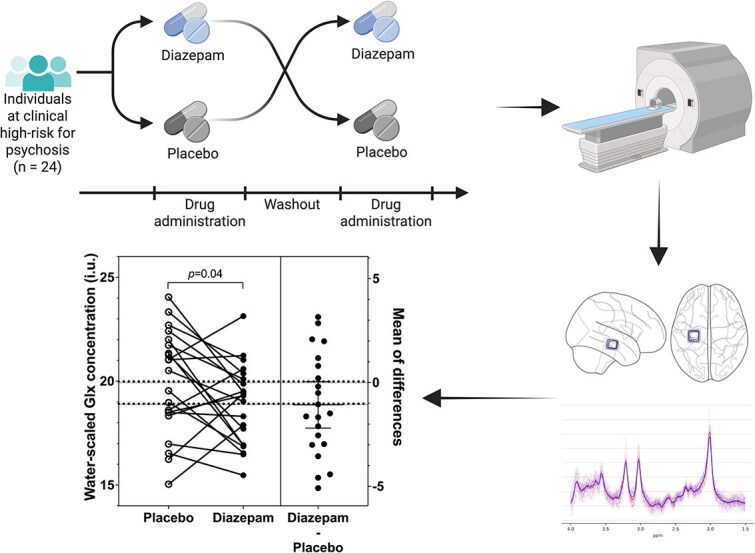

Significance Statement Psychotic disorders, such as schizophrenia, are often preceded by subtle changes in brain chemistry. In this study, we explored whether diazepam, a medication commonly used to treat anxiety, could influence brain chemistry in individuals at high risk for developing psychosis. Our findings revealed that a single dose of diazepam reduced levels of Glx (glutamate and glutamine)—key metabolites in excitatory neurotransmission—in a region of the brain involved in psychosis. These results suggest that diazepam may alter differences in brain chemistry linked to the development of psychosis, offering potential insights for early intervention strategies.

## INTRODUCTION

Individuals at clinical high risk for psychosis (CHRp) have a 27% risk of developing a psychotic disorder within 3 years of presentation.^1^ However, at present, there are no approved treatments for the prevention of psychosis in this population.^2^ Understanding the neurobiology of psychosis risk is critical for the development of novel preventive treatments, which could potentially benefit many young people who may develop poor long-term outcomes.^3^

Postmortem, preclinical, and in vivo clinical studies indicate that differences in the interplay between neuronal glutamatergic excitation and GABAergic inhibition play a role in psychosis. Postmortem studies in schizophrenia have reported reduced expression of GABAergic interneurons in the prefrontal cortex and hippocampus,^4,5^ and reductions in the GABA-synthesizing enzyme GAD67. Further studies have described reduced mRNA expression of the α_1_ subunit of GABA_A_ receptors (GABA_A_R) in pyramidal neurons in the dorsolateral prefrontal cortex, and elevations in the density of α_2_ subunits in prefrontal areas.^6^ The findings from in vivo positron emission tomography (PET) studies using tracers for α_1_-α _3_ and α_5_ subunit–containing GABA_A_R (GABA_A_ receptors with a benzodiazepine-binding site; GABA_A_-BZR) have been less consistent.^7^ One study using [^11^C]Ro15-4513, which has a preferential affinity for the α_5_ GABA_A_R subunit, reported reduced binding in the hippocampus of antipsychotic-free patients with schizophrenia.^8^ However, studies using more general GABA_A_-BZR ligands, such as flumazenil, have not identified differences in GABA_A_-BZR availability in the hippocampus in schizophrenia.^7^ Similarly, while one study found an increase of GABA_A_-BZR availability in the anterior cingulate cortex (ACC),^9^ most have not found evidence of altered GABA_A_-BZR availability in the ACC or other cortical regions.^7^ Notably, one study focusing on a cohort of CHRp individuals identified reductions in GABA_A_-BZR availability exclusively in the right caudate.^10^

More consistent findings have been evident from in vivo proton magnetic resonance spectroscopy (^1^H-MRS) studies of glutamate + glutamine (Glx) levels. In schizophrenia, Glx elevations have been consistently observed in the hippocampus.^11,12^ In individuals at CHRp, studies consistently report Glx increases in the medial prefrontal cortex (mPFC), including the ACC, as demonstrated through a meta-analysis.^11^ Individual studies additionally report elevations in striatal glutamate^13^ and Glx^14^ in CHRp individuals, with elevated glutamate in this region relating to the subsequent transition to psychosis.^15,16^ However, hippocampal Glx levels in CHRp individuals show a less consistent pattern. While some studies report increases,^16,17^ meta-analyses do not find hippocampal Glx changes,^11,12,18^ potentially due to the technical difficulties in Glx ^1^H-MRS imaging in the hippocampus. Glx elevations in schizophrenia have also been correlated with symptom severity: greater mPFC Glx has been associated with greater positive symptom severity, and greater medial temporal lobe Glx has been correlated with greater negative symptom severity.^19^

These findings align with preclinical evidence demonstrating that dysfunction of GABAergic interneurons in the ventral hippocampus can disrupt glutamatergic signaling and broader neural circuits. Studies in the methylazoxymethanol acetate (MAM) developmental rodent model found that GABAergic interneuron dysfunction in the ventral hippocampus leads to hyperactivity and increased glutamatergic outputs from this region.^20,21^ Through various downstream multisynaptic pathways, this mechanism is proposed to drive a hyperdopaminergic state in subcortical regions, which is linked to behavioral analogs of psychotic symptoms,^20,22^ mPFC changes related to cognitive symptoms, and alterations in the basolateral amygdala and ACC associated with negative symptoms.^20^ Repeated oral administration of diazepam, a positive allosteric modulator of the GABA_A_-BZR site, to MAM-treated rats during puberty prevented the subsequent development of dopamine neuron hyperactivity in the ventral tegmental area,^23^ normalized amygdala hyperactivity and anxiety-like behavior,^24^ and reduced the loss of GABAergic inhibitory interneurons in the ventral hippocampus.^24^ Independent work on another rodent model involving a genetic knockout of *Erbb4*, a schizophrenia susceptibility gene encoding a tyrosine kinase receptor involved in neuregulin signaling and critical for the maturation and function of fast-spiking interneurons,^25^ found that diazepam administration ameliorated schizophrenia-relevant deficits in prepulse inhibition.^26^ Our previous research in *Erbb4* conditional mouse mutants using translational in vivo neuroimaging revealed increased hippocampal cerebral blood flow and levels of hippocampal glutamine in this model.^27^

The present study aimed to examine the acute, mechanistic effects of diazepam on Glx levels in individuals at CHRp. We selected the ACC as the region of interest because previous research has consistently found elevated Glx levels in the ACC and not in the hippocampus in individuals at CHRp.^11^ Additionally, diazepam’s GABAergic effects in the hippocampus are hypothesized to modulate glutamatergic projections from the hippocampus to the ACC, leading to downstream reductions in glutamate release and Glx levels in the ACC. We hypothesized that diazepam would reduce ACC Glx levels in individuals at CHRp, putatively by increasing GABA binding to GABA_A_-BZR on pyramidal neurons, thereby reducing excitatory neuronal activity, glutamate release, and glutamate-glutamine cycling.

## METHODS

### Participants

Twenty-four individuals at CHRp aged 18-35 were recruited from the OASIS (Outreach and Support in South London) service.^28^ Based on data from previous ^1^H-MRS research at the same scanning site,^29^ our sample size was determined via a priori power calculation using G*Power version 3.1.9.7^30^ (power = 0.80, alpha = 0.05, two-tailed).

CHRp status was determined using the four positive subscales (unusual thought content [P1], non-bizarre ideas [P2], perceptual abnormalities [P3], and disorganized speech [P4]) of the Comprehensive Assessment of At-Risk Mental States (CAARMS).^31^ All the individuals were required to exhibit current attenuated psychotic symptoms, determined with a severity or frequency score ≥3 on P1-P4 of the CAARMS, in the presence or absence of genetic risk and deterioration syndrome or a history of brief limited intermittent psychotic symptoms. Participants fulfilling any of the following criteria were excluded from the study: (1) previous/current exposure to antipsychotic medication at any dosage, (2) diagnosis of a psychotic disorder, (3) diagnosis of a neurological disorder, (4) estimated IQ < 70 using the Wechsler Adult Intelligence Scale short version,^32^ (5) contraindication to MRI, (6) current exposure to drugs with potential GABAergic or glutamatergic effects (benzodiazepines, anticonvulsants, zopiclone, zolpidem, ketamine, opiates, atomoxetine, memantine, mood stabilizers) other than antidepressants, determined by participant self-reporting and urine drug testing on scanning days, and (7) pregnancy/breastfeeding, determined by urine pregnancy test. The study received ethical approval from the London–Bromley Research Ethics Committee (18/LO/0618). All the participants provided informed consent in accordance with the Declaration of Helsinki.

### Procedure

This experimental medicine study received full ethical approval from the UK National Health Service Research Ethics Committee and was conducted at King’s College London. It was deemed “not a Clinical Trial of an Investigational Medicinal Product” in accordance with the EU Directive 2001/20/EC but was nonetheless registered on ClinicalTrials.gov (NCT06190483). This was a randomized, double-blind, placebo-controlled crossover design study, by which participants were scanned with MRI twice, once under placebo (50 mg ascorbic acid; Crescent Pharma Ltd, Hampshire, United Kingdom) and once under a single dose of diazepam (5 mg; generic), separated by a minimum 3-week washout period. Female participants were mostly scanned with a 4-week interval (28 ± 3 days) to minimize metabolite fluctuations throughout the hormonal cycle^33,34^ (*n* = 9 scanned at four-week interval, *n* = 6 scanned at <25 or >31 days). Before the first scanning visit, an assessment visit was conducted to collect sociodemographic information, basic medical history, drug and cigarette use, the shortened Wechsler Adult Intelligence Scale–III^32^ to determine estimated IQ, and clinical measures (CAARMS positive and negative subscales,^31^ Hamilton Anxiety^35^ and Depression^36^ scales, and Global Functioning Role and Social scales^37^). CAARMS positive and negative composite scores were calculated as sums of subscale severity × subscale frequency (see the Supplementary Methods for score calculation).

Prior to scanning, participants were asked to abstain from alcohol, grapefruit-containing products, caffeine for 24 hours, and nicotine for 4 hours. A previous pharmacokinetic study^38^ demonstrated that food reduces and delays the peak plasma concentration of diazepam. Thus, to maximize the absorption rate of diazepam and ensure peak effects of diazepam during scanning,^38^ for morning scans (before 1:00 pm), participants were asked to fast from midnight onward, and for afternoon scans (after 1:00 pm), they were asked to eat a light breakfast before 10:00 am and fast onward. Oral diazepam or placebo capsules were administered with 200 mL of water 1 hour prior to scanning to capture the peak plasma window (1-1.5 hours^39^; average peak plasma time 1 hour postadministration for 5 mg diazepam^38,40^).

### Blinding, Randomization, and Allocation

A diazepam dose of 5 mg was selected on the basis that this produces significant clinical and pharmacokinetic effects without excessive sedation.^40^ To maintain blinding of participants, study staff, and radiographers, generic diazepam and placebo pills were removed from packaging and placed into an opaque gel capsule (Capsugel, Morristown, NJ, United States) a maximum of 1 hour prior to prescription collection by the Maudsley hospital outpatient pharmacy. Prescription was collected 30-45 minutes prior to dosing time.

A randomization list was generated by a researcher outside of the study team using a generalized Latin square (Williams Designs) to control for order and first-order carryover effects.^41^ Randomization numbers were allocated one of two possible treatment orders (AB or BA, where A = diazepam and B = placebo). Participants were allocated a sequential randomization number when the first-scan prescription was requested. Treatment order allocation was kept concealed from the study team at the Maudsley hospital outpatient pharmacy.

### MRI Acquisition

Participants were scanned using a General Electric (Chicago, WI, United States) MR750 3.0 T MR scanner with a 32-channel head coil at the Centre for Neuroimaging Sciences, Institute of Psychiatry, Psychology and Neuroscience, King’s College London. A *T*_1_-weighted inversion recovery spoiled gradient echo sequence (TE/TI/TR = 3.02/400/7.31 ms; flip angle = 11°; matrix = 256 × 256; FoV = 270; in-plane resolution = 1.05 × 1.05 mm^2^; slice thickness = 1.2 mm; 196 slices) was acquired for ^1^H-MRS voxel placement and voxel tissue content calculation.

**Figure 1:**
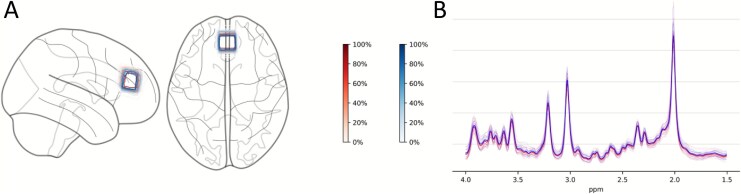
^1^H-MRS voxel placement and spectra overlap. (A) Normalized voxel placement for individual participants and conditions in the anterior cingulate cortex. Shading of contour lines on the glass brain and the color scales indicate the percentage of voxel overlap across participants within a condition, with darker red areas showing greater overlap and lighter shades indicating lower overlap. (B) Fitted spectra of individual participants and conditions and average spectra per condition in the anterior cingulate cortex. Red, placebo condition; blue, diazepam condition. Figure generated using MRS-voxel-plot^43^ with nilearn.^44^ ppm, parts per million.

^1^H-MRS spectra were acquired from an ACC voxel (20 × 20 × 20 mm^3^), placed according to previous studies,^29,42^ using point-resolved spectroscopy (PRESS; TE/TR = 30/3000 ms; flip angle = 90°; 96 transients) (Figure 1). Briefly, the center of the voxel was placed along the sagittal midline, 16 mm above the anterior portion of the genu of the corpus callosum, 90° to the anterior commissure to posterior commissure line. Water suppression was performed with the standard GE PROBE (proton brain examination) sequence, which utilizes a standard chemically selective suppression (CHESS) water suppression routine. For each spectrum, unsuppressed water reference spectra were acquired (16 transients) to perform eddy current correction and water scaling during spectral analysis. Shimming and water suppression were optimized by performing auto-prescan twice before each scan.

Spectra were pre-processed using the FID-A toolkit.^45^ This included multicoil combination, removal of bad averages, and frequency and phase drift correction. Subsequently, spectra were analyzed in LCModel 6.3-1N (see Figure S1 for sample spectrum). Water-suppressed spectra were fitted to a standard basis set included in LCModel, including 17 metabolites (l-alanine, aspartate, creatine [Cr], phosphocreatine [PCr], γ-aminobutyric acid, glucose, glutamine, glutamate [Glu], glutathione, glycerophosphocholine, phosphocholine, *myo*-inositol [mI], l-lactate, *N*-acetylaspartate [NAA], *N*-acetylaspartylglutamate, *scyllo*-inositol, and taurine), and acquired using PRESS with the same echo time (30 ms) and at the same field strength (3 T). To account for random variation in data fitting to basis set, a bootstrap analysis was performed. Linear combination modelling within LCModel was performed iteratively (50 modeling replicates). Due to the non-normal distribution of the replicate linear combination modeling output, median values were used for subsequent statistical analysis (sensitivity analysis using mean values was performed and is found in the online supplementary materials and results; Figure S2).

Initial quality control of spectra involved assessment of the signal-to-noise ratio (S/N) and spectral linewidth (full width at half-maximum, FWHM), and visual inspection. Spectra of FWHM > 2 standard deviations above the mean and S/N < 2 standard deviations (SDs) below the mean^46^ and data points with Cramér-Rao lower bound (CRLB) minimum variance > 20% were excluded. To correct water-scaled metabolite values for voxel tissue composition, segmentation was performed using Gannet 3.1 (Matlab 9.5.0; SPM 12; FSL 5.0.11) to attain the fraction of cerebrospinal fluid (CSF), gray matter (GM), and white matter (WM) per voxel per individual. Metabolites (M) were corrected for CSF according to the following formula: ${M}_{\mathrm{corr}}=M\times \frac{\mathrm{WM}+1.21\mathrm{GM}+1.55\mathrm{CSF}}{\mathrm{WM}+\mathrm{GM}}$. This formula assumes a water tissue concentration of 55 556 mmol L^−1^ in CSF, 35 880 mmol L^−1^ in WM, and 43 300 mmol/L in GM.^47^ The concentration of metabolites is expressed in institutional units (i.u.). The MRSinMRS checklist^48^ is reported in the online supplementary materials (Table S1).

### Statistical Analysis

Statistical analyses were performed using R (v4.2.2; https://www.r-project.org/). A significance threshold of *P* < .05 was set for all the analyses. Firstly, we assessed all within-group differences in data quality (FWHM, S/N) and voxel tissue composition (GM, WM, CSF) between the placebo and diazepam conditions via paired *t*-tests. Secondly, we analyzed changes in our a priori metabolite of interest (Glx) between the placebo and diazepam conditions using a weighted linear mixed effects model (lme4 package). Our primary mixed-effects model included participant ID as a random factor and treatment as a fixed-effect within-subject factor. As supplementary analyses, we performed two further models. To account for potential confounding treatment order, interscan interval, and data quality effects, treatment order and days between scans were added as covariates of no interest, and data quality (1−FWHM) as a weight factor. A second supplementary model investigated potential confounding effects of age, sex, current antidepressant treatment status,^49^ and cigarette use (cigarettes/day)^50–52^ by including these variables as covariates of no interest on top of the previous mixed-effects model analysis. No outliers were detected using Grubbs outlier detection.

Exploratory regression analyses investigated whether baseline clinical characteristics (CAARMS positive or negative composite scores) were related to Glx change (diazepam minus placebo) in the ACC. Both CAARMS subscale composite scores were entered into one multiple regression model. Potential confounding effects of age, sex,^53^ current antidepressant treatment status,^54^ and cigarette use^50^ on these associations were explored by adding these variables as covariates of no interest in a second model. Significance was set at *P* < .05, and multiple comparison corrections were not performed as these multiple regression models were exploratory.

## RESULTS

### Sample Characteristics

A total of 24 individuals at CHRp with a mean age ± SD of 24.1 ± 4.8 years were scanned twice. Demographic and clinical characteristics of the sample are presented in Table 1.

**Table 1:** Demographic and clinical characteristics

	**Variable**	**CHRp individuals (*n* = 24)**
**Demographics**	Age, mean ± SD years	24.1 ± 4.8
	Age range, years	18-32
	Sex, *n*, female/male	15/9
	Handedness, *n*, right/left	23/1
	Ethnicity, *n*, Asian/Black/Mixed/White	2/6/4/12
	IQ, mean ± SD	97.7 ± 21.6
**Clinical**	CAARMS positive composite, mean ± SD	46.4 ± 13.0
	CAARMS negative composite (*n* = 22), mean ± SD	29.4 ± 24.2
	HAM-A (*n* = 22), mean ± SD	17.1 ± 8.7
	HAM-D (*n* = 21), mean ± SD	13.9 ± 6.9
	GF:S current, mean ± SD	6.4 ± 1.5
	GF:R current, mean ± SD	6.1 ± 1.8
	Current antidepressant medication, *n*, yes/no	9/15
**Substance use**	Current cigarette use, *n*, yes/no	8/16
	Cigarettes/day in smokers, mean ± SD	1.8 ± 1.6
	Current cannabis use, *n*, yes/no	7/17

IQ, estimated intelligence quotient using the shortened Wechsler Adult Intelligence Scale; CAARMS positive composite, composite score (severity × frequency) of positive subscales (P1-4) on the Comprehensive Assessment of At-Risk Mental States; CAARMS negative composite, composite score (severity × frequency) of negative subscales (4.1-4.3) on the Comprehensive Assessment of At-Risk Mental States; HAM-A, Hamilton Anxiety Rating Scale; HAM-D, Hamilton Depression Rating Scale; GF:S, Global Functioning Social Scale; GF:R, Global Functioning Role Scale.

Participants were scanned with a mean ± SD of 30 ± 8 days of interscan interval (female participants: *M* ± SD = 28 ± 4 days; male participants: *M* ± SD = 33 ± 11 days). The range and mean ± SD of time from dosing to ^1^H-MRS scanning was 52-97 and 71 ± 9 minutes. All the participants completed both ACC ^1^H-MRS scans. Data quality parameters are presented in Table 2. No significant differences in spectral quality or voxel tissue composition between the diazepam and placebo conditions were observed. Due to spectral artifacts and quality control failure, two datapoints from the diazepam condition and one datapoint from the placebo condition were excluded. These excluded datapoints were from three different participants, who were not removed from the overall analysis due to incomplete datasets. No further data was excluded based on CRLB minimum variance (>20%).

**Table 2:** Water-scaled Glx levels and spectral quality data.

	**Anterior cingulate cortex (*n* = 24)**		
	Placebo	Diazepam	Statistics
**Glx levels, i.u.**	20.17 ± 2.49	18.99 ± 1.97	*t*(20.8) = −2.14, *P* = .04
**Glx CRLB, %**	5.39 ± 0.68	5.66 ± 0.67	*t*(23) = −1.41, *P* = .17
**FWHM, ppm**	0.03 ± 0.01	0.03 ± 0.01	*t*(23) = 0.01, *P* = .99
**S/N**	33.46 ± 4.92	33.19 ± 6.84	*t*(23) = 0.22, *P* = .82
**GM**	0.66 ± 0.05	0.66 ± 0.05	*t*(23) = −0.17, *P* = .86
**WM**	0.09 ± 0.03	0.09 ± 0.03	*t*(23) = 1.05, *P* = .30
**CSF**	0.25 ± 0.04	0.26 ± 0.04	*t*(23) = −0.52, *P* = .60

Means ± SDs are reported. Glx, glutamate + glutamine; i.u., institutional units; CRLB, Cramér-Rao lower bounds; FWHM, full width half-maximum; ppm, parts per million; S/N, signal-to-noise ratio; GM, gray matter fraction; WM, white matter fraction; CSF, cerebrospinal fluid fraction.

### Effect of Diazepam on ACC Glx Levels

**Figure 2:**
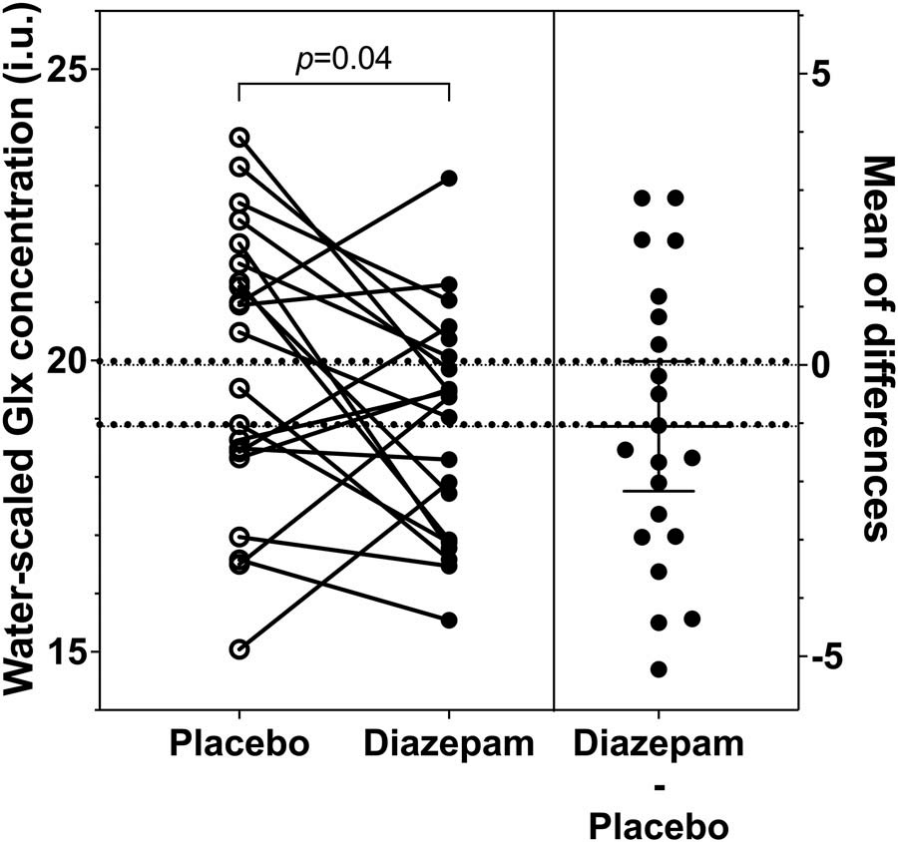
Glx levels in the anterior cingulate cortex during placebo and diazepam administration. Glx levels in the anterior cingulate cortex in individuals at clinical high risk for psychosis were reduced by acute diazepam administration. Individual Glx levels under the placebo and diazepam conditions are presented on the left and individual diazepam-induced Glx changes (diazepam–placebo) with mean and 95% CI are presented on the right. Incomplete datasets were removed for illustrative purposes. Glx, glutamate + glutamine.

In our primary analysis, ACC Glx levels were significantly lower under the diazepam condition (*M* ± SD = 18.99 ± 1.97) compared to the placebo condition (*M* ± SD = 20.17 ± 2.49; *t*(20.8) = −2.14, *P* = .04, *d* = 0.47; Table 2 and Figure 2). Similarly, ACC Glx levels were reduced under diazepam when accounting for treatment order, days between scans, and quality parameters (first supplementary model: *t*(20.7) = −2.11, *P* = .047, *d* = 0.46). Adding age, sex, antidepressant treatment, and cigarette use to the model (second supplementary model) rendered the observed treatment condition effect nonsignificant (*t*(20.9) = −2.04, *P* = .055, *d* = 0.45). None of the controlling variables had any significant effect in this model (see Table S2 for contribution of covariates in supplementary models). Female participants scanned at a 4-week interval (*n* = 9; 28 ± 3 days) had a mean diazepam-induced ACC Glx change of −1.27 (±2.39), and those not scanned within this interscan interval (*n* = 4; <25 or >31 days) of −0.69 (±2.45) (Figure S3).

### Exploratory Analyses

**Figure 3:**
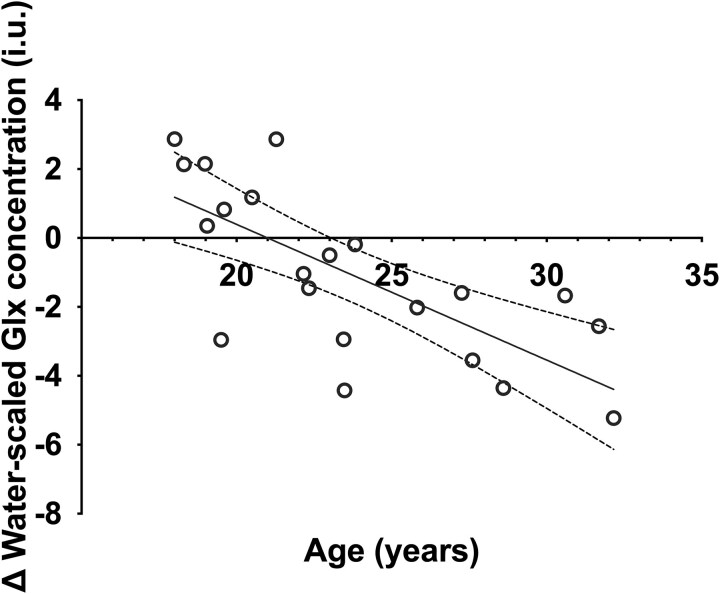
Diazepam-induced change in anterior cingulate cortex Glx over age of participants. The multiple regression model demonstrated a negative association with Glx change and age (diazepam Glx levels minus placebo Glx levels; *t*(12) = −4.36, *P* = .001). Interindividual diazepam-induced ACC Glx change was negatively associated with the age of participants. Glx, glutamate + glutamine.

Baseline CAARMS positive or negative composite scores were not associated with the diazepam-induced change in ACC Glx compared to placebo (model summary: *R*^2^ = 0.05, *F*(2, 16) = 0.44, *P* = .65). Adding sex, age, antidepressant treatment, and cigarette use as covariates rendered the regression model significant (model summary: *R*^2^ = 0.65, *F*(6, 12) = 3.73, *P* = .02; Figure 3). This was driven by a significant negative association between diazepam-induced change in ACC Glx levels and age (*t*(12) = −4.36, *P* = .001, *d* = 2.52). Further regressor statistics are reported in the Supplementary Results (Table S3).

## DISCUSSION

Our study revealed that an acute diazepam challenge modulated ACC Glx levels in CHRp individuals, which were significantly reduced compared to the ACC Glx levels of individuals on the placebo. There was an effect of age on these findings, by which the greatest diazepam-induced reductions in ACC Glx levels were observed in older CHRp individuals. These results provide preliminary support for the potential use of GABA-enhancing compounds as a therapeutic strategy to regulate brain excitation in early psychosis.

A previous meta-analysis of ^1^H-MRS studies in individuals at CHRp and in patients with a first episode of psychosis demonstrated alterations in Glx (glutamate + glutamine) and glutamine in the mPFC, including the ACC.^11^ Of note, a later meta-analysis which reviewed the ACC and the medial cingulate cortex separately based on reported voxel location found no significant Glx elevation in the ACC in CHRp groups compared to healthy controls (95% CI, −0.03 to 0.57, *P* = .08),^12^ highlighting that variation in ACC voxel placement likely contributes to discrepancies among individual studies. Nevertheless, in line with our hypothesis, diazepam reduced ACC Glx levels versus placebo in CHRp individuals. This suggests that elevated cortical glutamatergic function in this region and study population may be reduced by positive allosteric modulation of GABA_A_-BZR. Mechanistically, diazepam-enhanced inhibition may reduce glutamate release and glutamate-glutamine cycling,^55,56^ reflected as decreased Glx levels measured by ^1^H-MRS. Although glutamine is estimated to make up approximately 10% of the ^1^H-MRS Glx signal,^57^ it may be driving the observed ^1^H-MRS Glx changes, as it is primarily dedicated to neurotransmitter synthesis (≈70%-90%),^58^ specifically that of glutamate, and to a much lesser extent that of GABA.^59^ Future studies may tackle this question by ^1^H-MRS sampling in CHRp groups using higher-field (>4 T) scanners, where the glutamate and glutamine signal can be separated more reliably.^57^ For example, in a preclinical study using a 9.4 T scanner, we identified elevations in glutamine, but not glutamate, in *Erbb4* conditional mouse mutants, a genetic mouse model relevant to schizophrenia.^27^ Overall, together with our recent finding that a single dose of diazepam significantly normalized hippocampal hyperperfusion in the same sample of CHRp individuals,^60^ this evidence provides mechanistic support for the use of GABA-enhancing compounds to modulate neuroimaging-based phenotypes in individuals at CHRp, highlighting their potential as a therapeutic strategy to mitigate neurobiological changes in this patient group. These findings also align with previous preclinical work demonstrating that diazepam has a therapeutic effect on schizophrenia-relevant behavioral and electrophysiological phenotypes in genetic and neurodevelopmental rodent models.^23,24,26^ However, diazepam as well as other benzodiazepines may produce an unfavorable side effect profile due to its nonspecificity to general GABA_A_-BZR binding, causing drowsiness, fatigue, and confusion.^39^ More importantly, prolonged use can result in dependence and changes in receptor composition and function.^61^ However, more specific positive allosteric modulators of GABA_A_R avoid the sedation and dependence putatively mediated by effects on the α_1_ GABA_A_R subunit.^62^ In MAM rats, positive allosteric modulation of the α_5_ GABA_A_R^63^ and overexpression of the α_5_ subunit normalized hippocampal hyperactivity in adult rodents.^64^ Thus, compounds with higher GABA_A_R specificity, such as to the α_5_ GABA_A_R subunit, may have similar efficacy in humans without undesirable side effects.

Exploratory analysis suggested that diazepam-induced reductions in ACC Glx were not significantly associated with baseline symptom severity. However, we observed that age was a significant contributor to the model and was negatively associated with change in ACC Glx, such that younger participants exhibited smaller reductions or even increases in Glx under diazepam and older participants showed greatest Glx reductions under diazepam compared to placebo. A previous mega-analysis^65^ found that variability in Glx levels in patients with schizophrenia compared to healthy volunteers in the mPFC (including the ACC) increased with older age, suggesting greater dysfunctional regulation of glutamate levels with age. Due to the role of GABAergic transmission in the tight regulation of excitatory signaling,^66^ diazepam as a GABA-enhancing compound may have greater efficacy in more dysregulated states. Together, these findings suggest that GABA-enhancing compounds may induce greater modulation of brain excitatory metabolites in older individuals. As our sample was not originally statistically powered for these correlational analyses, these findings warrant replication in larger samples. However, this preliminary finding is consistent with an age-related decline in GABA inhibition,^67,68^ which may result in the greater efficacy of benzodiazepine.^69^ In contrast, preclinical studies using MAM-treated rats have demonstrated that chronic peripubertal diazepam administration prevented the development of schizophrenia-like phenotypes, suggesting that a chronic treatment paradigm may be required to achieve effects in younger individuals.

This study had some limitations. Firstly, as we did not include a comparator group of healthy control individuals, it is unknown whether ACC Glx levels were elevated in this CHRp cohort or whether the effects of diazepam are greater in individuals meeting CHRp criteria compared to healthy volunteers. However, elevations in ACC Glx in individuals at CHRp are a finding supported by meta-analytical evidence.^11,18^ Moreover, more than half of our participants were on antidepressants, reflective of the substantial real-world proportion of CHRp individuals who receive antidepressant treatment.^70^ Antidepressant treatment was previously shown to affect Glx levels in healthy individuals in several brain regions, although the ACC was not included and it was not a crossover design.^71^ As our sensitivity analysis adding age, sex, antidepressant treatment, and cigarette use to the model rendered the diazepam effect no longer significant, future studies should expand on whether this reflects compromised power after addition of further nuisance covariates, or confounding by antidepressants. Our mechanistic, experimental medicine study by acute single-dose drug administration focused on neuroimaging as its outcome measure. Future studies with a treatment course are warranted to enable assessing the effects of diazepam on positive, negative, and cognitive symptoms in CHRp individuals, who experience symptoms of subclinical frequency and severity as assessed over a longer time period (eg, over the previous month^72^), and correlate these with changes in Glx. Lastly, we did not measure diazepam plasma levels, preventing analysis of Glx changes relative to achieved drug dose. Future studies should incorporate blood sampling post-MRI to address this.

Overall, this study provides preliminary evidence that a single dose of the benzodiazepine diazepam reduces ACC Glx levels compared to placebo in CHRp individuals. This finding suggests a novel therapeutic mechanism which may be of benefit in individuals with psychosis vulnerability. The development of more selective GABA-enhancing compounds could allow similar regulation of brain excitation in people with psychosis risk while avoiding some of the unwanted side effects of less selective benzodiazepines.

## Supplementary Material

pyaf078_BENZOGAP_MRS_Manuscript_Supplement_v4_1

## Data Availability

The data underlying this article cannot be shared publicly for the privacy of the individuals who participated in the study. The data may be shared on reasonable request to the corresponding and senior author.
