# Supplementary material for: Diazepam modulates anterior cingulate glutamate levels in people at clinical high-risk for psychosis
Source: Int J Neuropsychopharmacol. 2025 Dec 12;29(2):pyaf078. doi: 10.1093/ijnp/pyaf078 (PMC12874875; doi:10.1093/ijnp/pyaf078)
Supplement: pyaf078_BENZOGAP_MRS_Manuscript_Supplement_v4_1 [file pyaf078_benzogap_mrs_manuscript_supplement_v4_1.docx]

## SUPPLEMENTARY MATERIALS

**TITLE: Diazepam modulates anterior cingulate glutamate levels in people at clinical high-risk for psychosis**

Amanda Kiemes^1^, Nicholas R. Livingston^1^, Paulina B. Lukow^1,2^, Samuel Knight^1^, Luke Jelen^1^, Thomas Reilly^3,4,5^, Aikaterini Dima^4^, Maria A. Nettis^1^, David J. Lythgoe^6^, Cecilia Casetta^4,5^, Alice Egerton^5^, Thomas Spencer^5^, Andrea De Micheli^5^, Paolo Fusar-Poli^5,7,8,9^, Anthony A. Grace^10^, Steven C. R. Williams^6^, Philip McGuire^3^, Cathy Davies^5^, James M. Stone^11^ & Gemma Modinos^1,12^

1. Department of Psychological Medicine, Institute of Psychiatry, Psychology and Neuroscience, King’s College London, London, UK
2. Institute of Cognitive Neuroscience, University College London, London, UK
3. Department of Psychiatry, University of Oxford, Oxford, UK
4. South London and Maudsley NHS Foundation Trust, London, UK
5. Department of Psychosis Studies, Institute of Psychiatry, Psychology and Neuroscience, King’s College London, London, UK
6. Department of Neuroimaging, School of Neuroscience, Institute of Psychiatry, Psychology and Neuroscience, King’s College London, London, UK
7. OASIS Service, South London and the Maudsley NHS Foundation Trust, London, United Kingdom
8. Department of Brain and Behavioural Sciences, University of Pavia, Pavia, Italy
9. Department of Psychiatry and Psychotherapy, Ludwig-Maximilian-University Munich, Munich, Germany
10. Departments of Neuroscience, Psychiatry and Psychology, University of Pittsburgh, Pittsburgh, PA, USA
11. Brighton and Sussex Medical School, University of Sussex, Brighton, UK
12. MRC Centre for Neurodevelopmental Disorders, King's College London, London, UK

### Supplementary Methods

#### Magnetic Resonance Spectroscopy

**Table S1.** MRSinMRS checklist

| 1. Hardware |  |
| --- | --- |
| a. Field strength [T] | 3 T |
| b. Manufacturer | General Electric |
| c. Model (software version if available) | MR750 |
| d. RF coils: nuclei (transmit/receive), number of channels, type, body part | 32-channel head coil |
| e. Additional hardware | N/A |
| 2. Acquisition |  |
| a. Pulse sequence | Point-resolved spectroscopy (PRESS) |
| b. Volume of Interest (VOI) locations | Anterior cingulate cortex (ACC) |
| c. Nominal VOI size [cm^3^, mm^3^] | 2 x 2 x 2 cm^3^ |
| d. Repetition Time (TR), Echo Time (TE) [ms, s] | TR 3000 ms, TE 30 ms |
| e. Total number of Excitations or acquisitions per spectrum  In time series for kinetic studies   1. Number of Averaged spectra (NA) per time-point 2. Averaging method (e.g. block-wise or moving average) 3. Total number of spectra (acquired / in time-series) | 96 acquisitions with an 8-step phase cycle. For the unsuppressed water, 16 acquisitions with an 8-step phase cycle. |
| f. Additional sequence parameters (spectral width in Hz, number of spectral points, frequency offsets)  If STEAM:, Mixing Time (TM)  If MRSI: 2D or 3D, FOV in all directions, matrix size, acceleration factors, sampling method | 5000 Hz, 4096 points, -2 ppm frequency offset |
| g. Water Suppression Method | Chemically selective suppression (CHESS) |
| h. Shimming Method, reference peak, and thresholds for “acceptance of shim” chosen | Automated B0 field mapping to < 7 Hz |
| i. Triggering or motion correction method  (respiratory, peripheral, cardiac triggering, incl. device used and delays) | N/A |
| 3. Data analysis methods and outputs |  |
| a. Analysis software | FID-A, LCModel 6.3-1N |
| b. Processing steps deviating from quoted reference or product | None |
| c. Output measure  (e.g. absolute concentration, institutional units, ratio) | Water-scaled (Estimated mM) |
| d. Quantification references and assumptions, fitting model assumptions | Default basis set provided in LCModel 6.3-1N (press_te30_3t_v3.basis) of 17 metabolites (L-alanine, aspartate, creatine (Cr), phosphocreatine (PCr), γ-aminobutyric acid, glucose, glutamine, glutamate (Glu), glutathione, glycero-phosphocholine, phosphocholine, myo-Inositol (mI), L-lactate, N-acetylaspartate (NAA), N-acetylaspartylglutamate, scyllo-inositol, and taurine) |
| 4. Data Quality |  |
| a. Reported variables  (SNR, Linewidth (with reference peaks)) | S/N: Placebo 33.46 ± 4.92; Diazepam 33.19 ± 6.84  FWHM (ppm as reported by LCModel): Placebo 0.03 ± 0.01; Diazepam 0.03 ± 0.01 |
| b. Data exclusion criteria | FWHM>2SD; S/N<2SD; CRLB>20%; visual inspection |
| c. Quality measures of postprocessing Model fitting (e.g. CRLB, goodness of fit, SD of residual) | CRLB of Glx: Placebo 5.39 ± 0.68; Diazepam 5.66 ± 0.67 |
| d. Sample Spectrum | Figure 1; Figure S1 |


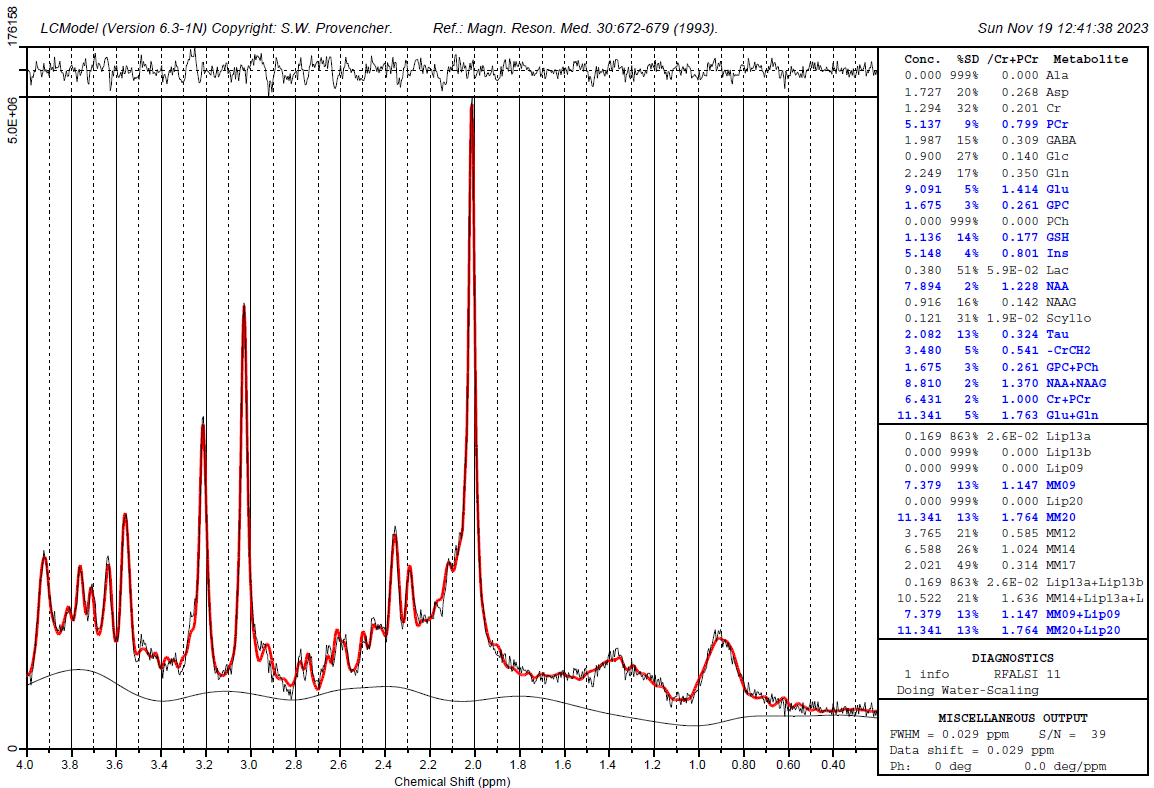


**Figure S1. Sample anterior cingulate cortex ^1^H-MRS spectrum**

#### CAARMS composite scores

CAARMS positive composite scores were calculated by multiplying the severity and the frequency of each positive domain subscale (unusual thought content, non-bizarre ideas, perceptual abnormalities, disorganized speech) and summing the products together. Similarly, the CAARMS negative composite score was ascertained using this method on the negative domain subscales (alogia, avolition/apathy, anhedonia).

#### Sensitivity Analysis

Our main analysis of Glx change between diazepam and placebo conditions used median scores from bootstrapped linear combination modelling. A sensitivity analysis was performed using mean value from replicate data fitting.

### Supplementary Results

#### Main Analysis

**Table S2.** Mixed effects model covariate statistics.

|  | t-value | df | p-value | d |
| --- | --- | --- | --- | --- |
| First supplementary model | | | | |
| *Treatment* | -2.110 | 20.749 | 0.047 | 0.46 |
| *Scanning order* | 0.565 | 19.837 | 0.579 | 0.13 |
| *Interscan interval* | 0.010 | 19.563 | 0.992 | 0.002 |
| Second supplementary model | | | | |
| *Treatment* | -2.037 | 20.857 | 0.055 | 0.45 |
| *Scanning order* | 0.403 | 16.514 | 0.692 | 0.10 |
| *Interscan interval* | 0.067 | 16.355 | 0.948 | 0.02 |
| *Age* | 0.620 | 16.858 | 0.544 | 0.15 |
| *Sex* | -0.866 | 16.549 | 0.399 | 0.21 |
| *Antidepressant treatment status* | 0.652 | 16.149 | 0.524 | 0.16 |
| *Cigarette use* | -0.633 | 15.713 | 0.536 | 0.16 |

#### Sensitivity Analysis

Linear mixed model using mean values from replicate data fitting analysis showed a significant effect of treatment condition, *t*(20.9) = -2.16, *p* = 0.04, d = 0.47 (Figure S2). Similarly to our main analysis, when accounting for treatment order, interscan interval and data quality, we found also found a significant effect of treatment condition, *t*(20.8) = -2.14, *p* = 0.04, d = 0.47. Lastly, when controlling for further covariates of no interest (age and sex), effect of treatment became non-significant (*t*(20.8) = -2.08, *p* = 0.051, d = 0.46).


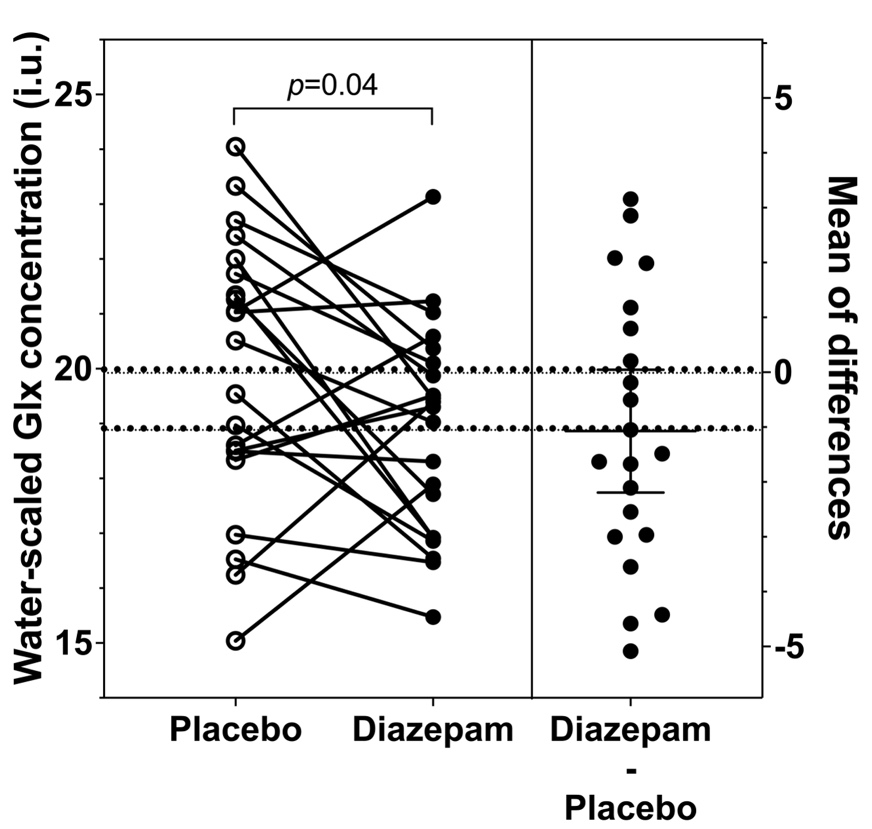


**Figure S2.** **Glx levels in the anterior cingulate cortex (sensitivity analysis using means from replicate linear combination modelling)*.*** Individual Glx levels during placebo and diazepam are presented on the left and individual diazepam-induced Glx change (diazepam – placebo) with mean and 95% CI are presented on the right. Incomplete datasets were removed for illustrative purposes. Glx, glutamate + glutamine.

**Table S3.** Regressors in multiple regression models.

|  | t-value | df | p-value | d |
| --- | --- | --- | --- | --- |
| Model 1 | | | | |
| *CAARMS positive composite score* | 0.476 | 16 | 0.641 | 0.24 |
| *CAARMS negative composite score* | -0.865 | 16 | 0.579 | 0.43 |
| Model 2 | | | | |
| *CAARMS positive composite score* | 0.144 | 12 | 0.888 | 0.08 |
| *CAARMS negative composite score* | 1.476 | 12 | 0.166 | 0.85 |
| *Age* | -4.360 | 12 | 0.001 | 2.52 |
| *Sex* | 0.616 | 12 | 0.549 | 0.36 |
| *Cigarettes per day* | -0.672 | 12 | 0.514 | 0.39 |
| *Antidepressant use* | 0.731 | 12 | 0.479 | 0.42 |

#### Female-only comparison of interscan interval groups


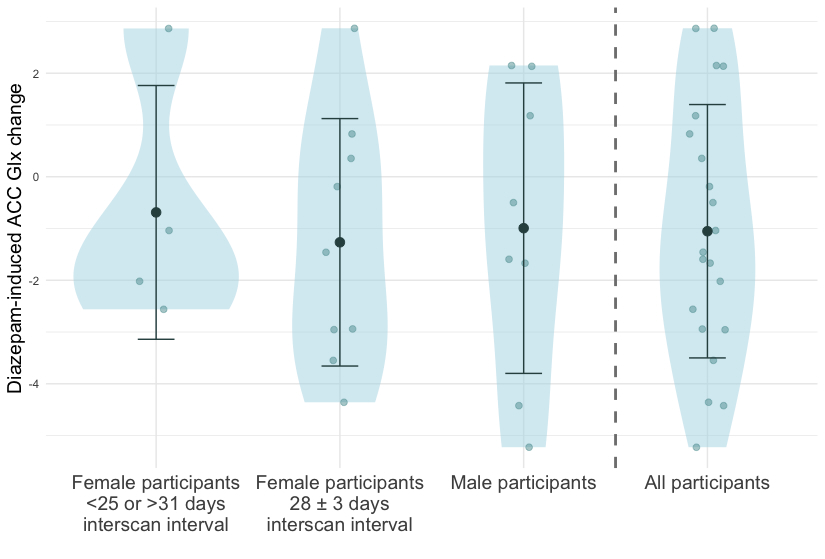


**Figure S3. Comparison of diazepam-induced ACC Glx change in female participants scanned within a four-week interscan interval or outside a four-week interscan interval**. Nine participants were scanned within a four-week interval (28 **±** 3 days; diazepam-induced ACC Glx change M **±** SD = -1.27 ± 2.39) and 6 were scanned outside of a four-week interval (<25 or >31 days; diazepam-induced ACC Glx change M **±** SD = -0.69 ± 2.45). The diazepam scan data point was excluded for 2 females scanned outside of the four-week interval, resulting in 4 female participants with ACC Glx change values in the non-four-week interscan interval group. For completeness, diazepam-induced ACC Glx change for male participants (M **±** SD = -0.99 ± 2.81) and all participants (M **±** SD = -1.05 ± 2.45) are presented. The placebo data scan was excluded for 1 male, resulting in 8 male participants with ACC Glx change values. Mean and standard deviations are displayed.
